# The association between resistance training volume load and session rating of perceived exertion in junior developmental female rugby league athletes

**DOI:** 10.1002/ejsc.12219

**Published:** 2024-11-28

**Authors:** Layne Flannery, Heidi R. Compton, Ben J. Dascombe, Millicent A. West, Josh L. Secomb

**Affiliations:** ^1^ Applied Sports Science and Exercise Testing Laboratory University of Newcastle Ourimbah New South Wales Australia; ^2^ School of Health Sciences Western Sydney University Campbelltown New South Wales Australia; ^3^ Active Living Research Program Hunter Medical Research Institute Callaghan New South Wales Australia

**Keywords:** external load, internal load, resistance training, training load, volume load

## Abstract

Field‐based team sports typically perform mixed‐modality training, incorporating both field‐ and resistance‐based sessions. As such, the availability of useful and reliable methods to monitor the internal and external training loads of all modalities is essential for planning effective training. Twenty‐one junior developmental female rugby league athletes (age: 17.5 ± 0.5 years, height: 167.7 ± 4.6 cm, body mass: 71.1 ± 12.9 kg, and training age: 2.3 ± 1.1 year) performed two to three resistance training sessions a week for 20 weeks (9 weeks preseason and 11 weeks in‐season). The volume load method and session rating of perceived exertion (sRPE) were used to quantify the external and internal load of the resistance training sessions, respectively. Volume load was categorised into either dynamic, plyometric, maximal or repeated efforts. Multiple linear mixed models were performed to determine whether significant relationships were present between the changes in volume load components and sRPE throughout the season. Significant relationships were identified between a decrease in sRPE, with associated increases in absolute and relative overall volume load (*T*_1,725.5_ = −2.1, *p* = 0.04; *T*_1,133.5_ = −2.2, *p* = 0.03), and relative dynamic (*T*_1,24.1_ = −8.4, *p* < 0.01) and lower‐body plyometric efforts (*T*_1,16.8_ = −17.2, *p* < 0.01). Conversely, significant relationships were observed between an increase in sRPE, with associated increases in relative lower‐body (*T*_1,20.3_ = 12.9, *p* < 0.01) and upper‐body repeated efforts (*T*_1,28.5_ = 9.7, *p* = 0.03) as well as relative upper‐body plyometric (*T*_1,71.1_ = 2.9, *p* = 0.01) and maximal efforts (*T*_1,75.3_ = 3.4, *p* < 0.01). These findings highlight the practicality of the volume load method for planning and monitoring resistance training in field‐based team sport athletes, providing useful data for the planning of specific exercises within the in‐season training week.

## Highlights

- The volume load method is a practical tool for monitoring and planning resistance training due to the significant relationships observed between changes in volume load components with alterations in sRPE throughout a competitive season.
- Increases in absolute and relative overall volume loads, as well as relative dynamic and lower‐body plyometric efforts, were associated with significantly lower perceived difficulty (sRPE).
- A greater volume of lower‐body and upper‐body repeat efforts as well as upper‐body plyometric and maximal efforts had a significantly higher psychophysiological perception of training intensity.
- Practitioners should consider implementing the volume load method for monitoring and planning resistance training sessions in field‐based team sports, calculating the associations between resistance training types and perceived difficulty within their cohort, to facilitate better in‐season planning and prescription.

## 1 INTRODUCTION

Training load is recognised as the cumulative amount of stress placed on an individual from a variety of training sessions over a specific period of time (Gabbett et al., 2014). Numerous methods are available to quantify this load, with the two categories of “external training load” and “internal training load” commonly referred to in sport (Halson, 2014). External training load quantifies the volume of work completed by an athlete and is measured independent of their internal characteristics (Wallace et al., 2009). Conversely, the internal training load involves measuring the athletes' relative physiological and psychological stress imposed by the training (Cummins et al., 2013; Impellizzeri et al., 2005; Rossi et al., 2017, 2019; Scott et al., 2016). When combined, these measures provide an indication of the athletes' training volume capacity and ability to cope with imposed stress (Halson, 2014). However, without practically useful methods to calculate the external and internal loads of all training modalities, practitioners will not be able to effectively plan and adjust training to optimise the preparation of athletes.

The primary aim of training load monitoring is to maximise competitive performance and reduce the opportunity for injuries (Booth et al., 2018; West et al., 2021). For example, excessive training loads with inadequate recovery may lead to suboptimal performance and an increased risk of injury due to heightened fatigue (Lambert et al., 2010; Vanrenterghem et al., 2017). Conversely, insufficient training loads can also result in suboptimal performance as the stimulus may not be sufficient to elicit the required adaptations, resulting in the athletes being unprepared to meet the physical and physiological demands of their sport (Lambert et al., 2010; Vanrenterghem et al., 2017). In field‐based team sports, practitioners and coaches need to carefully prescribe the appropriate volume and balance of both resistance‐ and running‐based training to optimise the athletes' preparedness (Gabbett, 2016). This is particularly crucial for some female field‐based team sports, such as rugby league, where the athletes are often considered semi‐professional, requiring them to balance the competing demands of work, social life, training and match play. The need to manage this variety of stressors highlights the importance of monitoring external load and the athletes' response to these. However, accurate calculation of the external training loads of these diverse modalities can be challenging due to difficulty in employing practically useful tools that are related to the internal loads (Scott et al., 2016).

To plan and monitor the external training load of running‐based training, practitioners typically analyse the total distance covered, high‐speed running distance and sprint and acceleration efforts (Conte et al., 2022; Cummins et al., 2013). Importantly, the session rating of perceived exertion (sRPE) is regarded as a valid and reliable internal training load measure. It has been significantly associated with external load measures in running‐based training and is more generalisable across training modalities compared to other measures such as heart rate (Day et al., 2004; Foster et al., 2001; Minganti et al., 2010). In contrast, for resistance‐based training prescription, a variety of methods exist to quantify the external training load, such as the repetition method, velocity monitoring or typically the volume load method, which is calculated as follows: volume load = sets × reps × load lifted (kg) and is more representative of the total work completed by the athlete (Scott et al., 2016). However, unlike running‐based training, limited research has related these external resistance‐based training load methods to internal load measures (Alexiou et al., 2008; Conte et al., 2022; Foster et al., 2001; Impellizzeri et al., 2004; McLaren et al., 2018).

Previous research indicates that resistance‐trained males and females report significantly different perceptions of physiological and psychological stress when performing a single session of force‐, velocity‐, or hypertrophy‐focused exercises (Day et al., 2004; Egan et al., 2006; Sweet et al., 2004). However, in field‐based team sports, practitioners typically utilise concurrent periodisation of resistance training and focus more on the adaptations that can be elicited throughout a competitive season (Aspe et al., 2022). To the best of our knowledge, no previous research has investigated the relationships between changes in resistance training volume load and sRPE in female field‐based team sport athletes. Additionally, the effect of altering the contribution of the various resistance training types to the overall volume, on changes in sRPE has not yet been determined. Therefore, the aims of this study were to determine the associations between changes in resistance training volume load and sRPE in female junior rugby league athletes throughout a competitive season and to identify which resistance training types demonstrated the greatest associations with reported sRPE.

## 2 MATERIALS AND METHODS

### 2.1 Study design

To explore the associations between changes in resistance training volume load and sRPE and identify the specific resistance training types with the greatest associations to reported sRPE, junior developmental female rugby league athletes performed two to three resistance training sessions each week for a 20‐week period. This period consisted of an 8‐week preseason (two 4‐week training blocks separated by a 1‐week break due to Christmas) and an 11‐week in‐season (a 9‐week regular season and 2‐week finals series) from November 2020 to April 2021. Training sessions were performed on a Monday, Wednesday and Friday throughout the training period, and during the in‐season competition, matches were played on a Saturday. During some weeks, the Monday training session was cancelled due to reasons such as athlete management decisions and public holidays. A total of 23 preseason and 29 in‐season sessions were completed by all athletes, each consisting of a resistance training component, immediately followed by a running‐based field session.

The resistance training sessions incorporated a combination of exercises with various intended outcomes such as enhanced power, maximal strength or hypertrophy development; however, the primary emphasis changed between training blocks. Specifically, during the first 4‐week preseason block, there was an emphasis on general strength and hypertrophy through a greater overall volume load. Conversely, in the second 4‐week preseason block, the focus was to increase maximal strength with a greater prescribed intensity in the main lifts, whilst maintaining a high‐volume load for the accessory exercises. During the in‐season, which consisted of two 4‐week blocks and one 3‐week block, a greater emphasis was placed on the improvement or maintenance of power and maximal strength. Each athlete was familiarised with the Borg CR‐10 scale prior to the start of the season and provided a verbal rating of perceived exertion (RPE) in isolation, approximately 20 min following the conclusion of each training session (Borg, 1998). Session length was recorded at the end of each session and was the same for all athletes, as the share gym space with strict time limits required all athletes to start and finish simultaneously. This rating was subsequently converted to an sRPE load by multiplying the RPE by the total duration of the training session in minutes, which was recorded by the lead researcher (LF) at the completion of each session.

### 2.2 Subjects

Twenty‐one junior developmental female rugby league athletes (age: 17.5 ± 0.5 years, height: 167.7 ± 4.6 cm, body mass: 71.1 ± 12.9 kg and training age: 2.3 ± 1.1 yr) volunteered to participate in the study. All athletes were recruited from the same rugby league team that competed in a state‐based competition and were free of any pre‐existing medical conditions that could restrict their ability to complete all training. Ethical clearance was obtained through a university ethics committee, and prior to the commencement of the study, all athletes were provided with detailed information prior to providing written informed consent. For athletes under 18 years of age, written informed consent was provided by their parent or guardian.

### 2.3 Procedures

One week prior to the commencement of preseason, all athletes were provided with information regarding the structure of the training sessions and expectations associated with attendance. Additionally, during the information session, the athletes were asked to self‐report the number of years and months that they had been undertaking resistance training prescribed by an accredited strength and conditioning practitioner, which was recorded as their resistance training age. Furthermore, the athletes' body mass index was assessed to the nearest 0.1 kg, using calibrated dual‐platform force plates (ForceDecks; VALD, Brisbane, Australia), which were used for the relative volume load calculations.

To quantify the external load of the resistance training sessions, the volume load method was utilised (volume load = sets × reps × weight lifted [kg]) (Scott et al., 2016). All training sessions were individually prescribed by an accredited strength and conditioning practitioner, using a custom Excel spreadsheet (Microsoft, Redmond, USA) that was automated to calculate the volume load of each exercise. Additionally, a code was applied to each exercise to categorise the resistance training type, based on those outlined in Zatsiorsky et al. (2020):

- Dynamic effort: a whole‐body movement with the intent of maximising the velocity of movement with a large external load, that is, a near‐maximal effort power clean.
- Plyometric effort: a whole‐body or upper‐body movement with the intent of maximising velocity of the movement with bodyweight (BW) only or a low external resistance load (<10 kg).
- Maximal effort: a movement with the intent of overcoming a large resistance to increase maximal force‐generating capabilities of the musculoskeletal system. These exercises were prescribed for sets of six repetitions or less.
- Repeated effort: a movement prescribed for sets of seven repetitions or greater, and with a low to moderate load to elicit an adaptation for muscular hypertrophy or endurance.

The resistance training sessions were quantified for both absolute (kg) and relative (kg·BW^−1^) overall loads, as well as analysed based on resistance training type and body region (whole body, upper body and lower body). Regarding quantification of specific exercises, for dynamic efforts and maximal efforts only the external load, with the absence of BW, was used in calculations. However, for plyometric effort exercises the athlete's BW plus any additional small external resistance were used for the calculation of volume load. The inclusion of BW for the plyometric efforts, but not dynamic efforts or maximal efforts, was performed to simplify the reproducibility of these methods for researchers and practitioners. For repeated effort exercises, where only the athlete's BW was used, set percentages of this BW were applied for calculating the resistance load. For example, the following percentages were used: push‐ups on toes (70%BW), push ups on knees (50%BW), front plank (30%BW), bilateral wall squat hold (30%BW), Nordic hamstring curl (50%BW), TRX 45° row (70%BW), single leg (SL) squat to a box (70%BW), SL squat on a box (100%BW), pull‐ups (100%BW) and inverted row (50%BW). These calculations were derived from the authors' (LF, JS) anecdotal evidence, and as the subsequent statistical analysis considered the individual athletes as an effect, this approach was deemed more appropriate for quantifying the volume load of each exercise.

### 2.4 Statistical analysis

To assess the relationships between the changes in volume load and sRPE, multiple linear mixed models were used. To account for the individual effect of volume load on sRPE and the repeated measures design of the study, a random intercept and slope structure were applied with the individual athletes included as random effects. Prior to analysis, both the independent (volume load) and dependent (sRPE) variables were log‐transformed, to allow for interpretation of results in terms of a percentage change (increase or decrease) in sRPE for every 1% increase in volume load. For practical interpretation, results were presented as a fixed 10% increase in various volume load variables and the associated effect on the sRPE. To calculate this associated effect, the following calculation was used; 1.1 to the power of the coefficient, subtracted by 1 and multiplied by 100. For example, if the calculated coefficient is 0.57, the formula was as follows: (1.1^0.57^–1) * 100 = 5.61%, representing the associated percentage change in sRPE with a 10% increase in the volume load variable. All analyses were conducted in R Studio (v 2022.12.0) and statistical significance was set at *p* ≤ 0.05.

## 3 RESULTS

The mean (±SD) of the session duration, sRPE and various volume load variables for a resistance training session in the preseason and in‐season are presented in Table 1. Additionally, the relationships between the change in volume load variables and the change in sRPE are displayed in Table 2. Significant inverse relationships were observed between a decrease in the reported sRPE with an associated increase in absolute and relative volume load, dynamic efforts and lower‐body plyometric efforts. More specifically a 10% increase in the volume load of dynamic efforts and lower‐body plyometric efforts were associated with a decrease in sRPE of 3% and 7%, respectively. Conversely, significant positive relationships were identified between an increase in sRPE, with an increase in the volume of lower‐body repeated efforts, and upper‐body plyometric, repeated and maximal efforts. A 10% increase in the contribution of the volume load of these resistance training types was associated with a change in sRPE of approximately 1%–2%.

**TABLE 1:** Mean ± standard deviation of the session rating of perceived exertion (sRPE) and the absolute and relative overall volume load (VL) and resistance training type components for the junior developmental female rugby league athletes (*n* = 21).

	Preseason	In‐season
Average session length (min)	54.1 ± 9.3	52.5 ± 10.8
Average session intensity (RPE)	5.7 ± 1.8	6.0 ± 1.4
sRPE (AU)	316 ± 120	332 ± 112

	Absolute (kg)	Relative (kg·kg·BM^−1^)	Absolute (kg)	Relative (kg·kg·BM^−1^)
Overall volume load	5181 ± 1345	73.3 ± 16.8	4516 ± 1440	64.1 ± 18.8
Dynamic efforts	616 ± 356	8.9 ± 5.2	495 ± 228	7.2 ± 3.7
Upper‐body volume load				
Plyometric efforts	299 ± 171	2.3 ± 2.9	282 ± 140	1.3 ± 2.3
Maximal efforts	1000 ± 328	14.1 ± 4.0	1712 ± 594	24.1 ± 7.8
Repeated efforts	1513 ± 796	21.4 ± 11.2	1706 ± 1023	24.2 ± 14.0
Lower‐body volume load				
Plyometric efforts	1737 ± 1445	24.6 ± 19.5	1619 ± 1764	23.0 ± 24.1
Maximal efforts	1130 ± 283	16.1 ± 4.2	1425 ± 350	20.4 ± 5.7
Repeated efforts	1947 ± 1323	27.5 ± 18.0	1006 ± 982	14.2 ± 13.6

**TABLE 2:** Linear mixed model output of the relationship between change in each volume load variable and the change in session rating of perceived (sRPE) load.

	Mixed model interpretation					Practical interpretation
	Estimate	Standard error	Degrees of freedom	T Statistic	*p*‐value	Associated % change in sRPE
Absolute						
Overall VL	−0.10	0.05	725.5	−2.1	0.04	−0.9
DE	−0.78	0.09	8.3	−8.3	<0.01	−7.1
LB PE	−0.32	0.02	17.1	−17.2	<0.01	−3.1
LB ME	−0.04	0.06	319.2	−0.8	0.45	−0.4
LB RE	0.18	0.01	20.2	13.2	<0.01	1.8
UB PE	0.14	0.05	223.8	3.0	<0.01	1.3
UB ME	0.09	0.03	41.4	3.1	<0.01	0.9
UB RE	0.22	0.02	406.4	9.9	<0.01	2.1
Relative						
Overall VL	−0.11	0.05	133.5	−2.2	0.03	−1.0
DE	−0.73	0.09	24.1	−8.4	<0.01	−6.7
LB PE	−0.32	0.02	16.8	−17.2	<0.01	−3.0
LB ME	−0.04	0.05	17.8	−0.7	0.48	−0.4
LB RE	0.18	0.01	20.3	12.9	<0.01	1.8
UB PE	0.13	0.05	71.1	2.9	0.01	1.3
UB ME	0.09	0.03	75.3	3.4	<0.01	0.9
UB RE	0.22	0.02	28.5	9.7	<0.01	2.2

*Note*: The practical interpretation outlines the expected change in sRPE based upon a 10% increase in the VL variable.

Abbreviations: DE, dynamic effort; LB, lower‐body; ME, maximal effort; PE, plyometric effort; RE, repeat effort; UB, upper‐body.

## 4 DISCUSSION

The primary purpose of this study was to explore the associations between the changes in resistance training volume load and sRPE throughout a season, thereby identifying the specific resistance training types with the greatest associations to the reported sRPE. Interestingly, significant inverse relationships were identified between a decrease in the reported sRPE, with associated increases in absolute and relative overall volume load, highlighting the importance of analysing the individual contribution of the specific resistance training types on sRPE. Specifically, dynamic efforts and lower‐body plyometric efforts displayed significant inverse relationships with sRPE. Conversely, significant relationships were observed between an increase in sRPE, with associated increases in the volume load for lower‐body and upper‐body repeated efforts, as well as upper‐body plyometric efforts and maximal efforts. Together, these data suggest that an increase in the contribution of dynamic efforts and lower‐body plyometric efforts to the overall volume load was associated with reduced psycho‐physiological perception of training intensity, whereas a greater contribution of lower‐body and upper‐body repeated efforts and upper‐body plyometric efforts and maximal efforts were perceived as higher intensity training by the athletes. The volume load method appears to be a practically useful tool for monitoring and planning resistance training in junior female rugby league athletes. However, it appears to be the intricate combination of prescribed resistance training types that will be important for practitioners to plan as opposed to merely calculating the overall volume load. As such, this study has important implications for training prescription in field‐based team sport athletes, particularly regarding the specific timing and emphases of resistance training type prescription throughout a competitive season and within a training week.

The initially surprising inverse relationship observed between increases in both absolute and relative overall volume load with a reduction in sRPE contrasted past data reported significant positive relationships between these measures in well‐trained adult males (age: 25.0 ± 4.0 years) (Genner et al., 2014). However, given the athletes in this study had a lower age (17.5 ± 0.5 years) and likely lesser physical maturity, sessions with a higher overall volume load were typically accumulated with lower relative intensity (percentage of one‐repetition maximum [%1RM]) loads that were prescribed for higher repetition sets. For example, for an athlete with a 1RM back squat of 120 kg, 4 sets of 5 repetitions at 85%1RM (100 kg) equates to a VL of 2000 kg, whereas 3 sets of 12 repetitions at 65%1RM (78 kg) equates to 2808 kg. Previous research supports this suggestion, with both general population males and females, and trained athletes, reporting a significantly higher RPE with greater relative intensity exercises (low repetition sets with higher %1RM) when compared to lower intensity exercises (more repetitions with a lower %1RM) (Day et al., 2004; Sweet et al., 2004). Similarly, Hiscock et al. (2015) identified that in volume load‐matched training sessions, a greater relative intensity (70%1RM) of exercises was associated with significantly greater sRPE than lower relative intensity (40%1RM). Therefore, the prescription strategy used in this study appears to explain why these higher overall volume load sessions were perceived as lesser psycho‐physiological difficulty by the athletes. Additionally, this highlights the importance for practitioners to calculate the volume load contribution of each resistance training type as well as the need for future research to explore similar questions and incorporate additional methods to assess muscle damage.

Significant positive relationships were observed between changes in the volume load of upper‐body maximal efforts, lower‐body repeated efforts and upper‐body repeated efforts with changes in sRPE. Crucially, the upper‐body maximal efforts prescribed in this study consisted of sets at a high relative intensity (>80%1RM), whereas the repeated efforts were typically prescribed with the intent of creating a stimulus for increased muscular hypertrophy (moderate relative intensities [60%–80%1RM], with sets of 7–15 repetitions performed to near momentary failure). Previous research has noted that this type of repeated effort prescription imposes substantial physiological and metabolic demands, leading to greater internal load perception (Martorelli et al., 2021). Similarly, Dos Santos et al. (2021) identified that well‐trained females performing sets of parallel squats reported significantly greater sRPE for sets completed to momentary failure compared to those that did not evoke momentary failure. Together, this suggests that either increased relative intensity of resistance training or training with moderate intensities to near momentary failure, even with a potentially lower overall volume load, were associated with greater perceived difficulty by the athletes in this study. Therefore, practitioners may consider prescribing this type of training more frequently during the preseason or earlier in the week during the in‐season, to reduce the perception of fatigue leading to a competition match.

Interestingly, an increase in the volume load of dynamic efforts and lower‐body plyometric efforts were related to significant decreases in the reported sRPE. This aligns with the previous research of Egan et al. (2006) and Martorelli et al. (2021), which identified that velocity‐focused prescription evoked significantly lower sRPE than both hypertrophy‐ and maximal strength‐focused prescription in resistance‐trained men. In this study, dynamic efforts and lower‐body plyometric efforts were prescribed to maximise movement velocity and minimise excessive fatigue, likely explaining the associated reduction in perceived psycho‐physiological difficulty. Specifically, all exercises were prescribed for a maximum of 5 repetitions per set, as research suggests that power output significantly diminishes following the seventh repetition of a velocity‐based exercise (Baker et al., 2007). Importantly, a 10% increase in dynamic effort volume load was associated with an approximately 7% decrease in sRPE, representing the largest magnitude associated change in perceived difficulty in this study. Consequently, for phases of the season or training week where practitioners want to minimise perceived difficulty, such as when tapering during the season, peaking before a match or days involving mixed‐modality training sessions, increasing the contribution of dynamic efforts and lower‐body plyometric efforts to the overall volume load will likely be beneficial. However, each individual athlete will have different preferences for the external and internal resistance training load to optimise their preparation to perform. Therefore, practitioners should acquire this information and utilise it when planning the weekly structure of resistance training sessions in‐season.

In contrast, the contribution to the volume load of upper‐body plyometric efforts and maximal efforts were associated with significantly greater perceived difficulty in this study. This data is in agreement with that of Day et al. (2004), which reported higher RPE for the bench press compared to a back squat at the same relative intensities (70% and 90% 1RM). The increased perception of difficulty for upper‐body exercises compared to the lower‐body is likely due to the lower cross‐sectional area of the muscles that contribute to the production of force in these exercises compared to the lower‐body exercises (Chilibeck et al., 1997). Although there is conflicting evidence regarding the recovery rate of the upper‐body musculature following resistance training (Baechle et al., 2008; Korak et al., 2015), the data from this study suggests that practitioners should consider prescribing the majority of upper‐body plyometric efforts and maximal effort volume load earlier in the training week, particularly for athletes who may experience negative psychological match preparedness from performing higher perceived difficulty training closer to a competition match.

The limitations of this study were that it consisted of athletes who were recruited from a single team, with these athletes categorised as junior developmental female rugby league athletes. Consequently, the applicability of these findings may be limited in terms of broader contexts. Specifically, the outcomes could be influenced by the specific characteristics of the junior developmental cohort studied, including factors such as their sex, training age and the typical training practices of the sport. Together, these may have influenced the associations observed in this study between external and internal load measures. Thus, future investigations should explore similar research questions in a variety of populations and determine whether these relationships vary between phases of the season, particularly when athletes may be carrying injuries and experiencing additional stress and anxiety during the in‐season. Furthermore, incorporating markers of neuromuscular fatigue and alternate methods to quantify resistance training exercise type, such as velocity‐based measurements, appears warranted.

## 5 CONCLUSIONS

The significant relationships observed between the change in volume load components and the associated alteration in sRPE emphasises the usefulness and importance of the volume load method for monitoring and prescribing resistance training in female rugby league athletes. Importantly, examining the overall volume load in isolation may not provide an accurate representation of the perceived difficulty of the session, making it crucial for practitioners to categorise and calculate the volume load of the various resistance training types within a training session. Crucially, the data of this study highlighted that the different volume load components elicited distinct differences in perceived psycho‐physiological difficulty. Specifically, increases in volume load for lower‐body and upper‐body repeated efforts, as well as upper‐body plyometric efforts and maximal efforts were perceived with greater psycho‐physiological difficulty. Conversely, a greater contribution of dynamic efforts and lower‐body plyometric efforts to the overall volume load was perceived with lower difficulty. Therefore, practitioners should consider prescribing training with a greater focus on dynamic efforts and lower‐body plyometric efforts when seeking to reduce the athletes' perceived difficulty of the training, such as when tapering and peaking for competition. Conversely, a higher proportion of volume load from repeated efforts and upper‐body maximal and plyometric efforts should be prioritised early in the training week and during preseason. However, practitioners should investigate the relationships between the volume load components and sRPE within their specific athlete cohorts and with consideration of each athlete's preference for training preparation, to better individualise training to optimise their physical and psychological preparedness for competition.

## CONFLICT OF INTEREST STATEMENT

The authors report there are no competing interests or financial relationships to declare.

## ETHICS APPROVAL STATEMENT

Ethical approval was granted by the University of Newcastle Human Ethics Committee (H‐2020‐0072), and all athletes or their guardians provided informed consent prior to the commencement of the study.

## Data Availability

The associated data from this study are available upon reasonable request.
